# Effects of Co-Modification by Extrusion and Enzymatic Hydrolysis on Physicochemical Properties of Black Wheat Bran and Its Prebiotic Potential

**DOI:** 10.3390/foods12122367

**Published:** 2023-06-14

**Authors:** Chunli Kong, Caiping Duan, Shunzhi Zhang, Rui Liu, Yuanlin Sun, Sumei Zhou

**Affiliations:** 1 School of Food and Health, Beijing Engineering and Technology Research Center of Food Additives, Beijing Advanced Innovation Center for Food Nutrition and Human Health, Beijing Technology and Business University, Beijing 100048, China; kongc0606@btbu.edu.cn (C.K.);; 2 Department of Life Sciences, Yuncheng University, Yuncheng 044000, China; 3 Shanxi Technology Innovation Center of High Value-Added Echelon Utilization of Premium Agro-Products, Yuncheng University, Yuncheng 044000, China

**Keywords:** black wheat bran, extrusion, enzyme, water extractable arabinoxylan, fermentation

## Abstract

Black wheat bran (BWB) is an important source of dietary fiber (DF) and phenolic compounds and has stronger nutritional advantages than ordinary WB. However, the low content of soluble dietary fiber (SDF) negatively influences its physicochemical properties and nutritive functions. To obtain a higher content of SDF in BWB, we evaluated the impact of co-modification by extrusion and enzymes (cellulase, xylanase, high-temperature α-amylase, and acid protease) on water extractable arabinoxylan (WEAX) in BWB. An optimized co-modification method was obtained through single-factor and orthogonal experiments. The prebiotic potential of co-modified BWB was also evaluated using pooled fecal microbiota from young, healthy volunteers. The commonly investigated inulin served as a positive control. After co-modification, WEAX content was dramatically increased from 0.31 g/100 g to 3.03 g/100 g (*p* < 0.05). The water holding capacity, oil holding capacity, and cholesterol adsorption capacity (pH = 2.0 and pH = 7.0) of BWB were increased by 100%, 71%, 131%, and 133%, respectively (*p* < 0.05). Scanning electron microscopy demonstrated a looser and more porous microstructure for co-modified BWB granules. Through in vitro anerobic fermentation, co-modified BWB achieved a higher content of *Bifidobacterium* and *Lactobacillus* than inulin fermentation. In addition, co-modified BWB induced the highest butyric acid production, indicating high potential as prebiotics. The results may contribute to improving technologies for developing high-fiber-content cereal products.

## 1. Introduction

Wheat bran (WB) is the major by-product of wheat during the milling process and accounts for about 25% of the total weight [1]. The biomass of WB can be estimated at around 150 million tons per year [2]. The WB fraction refers to the outer segment of the kernel and normally contains pericarp, testa, hyaline, aleurone, and endosperm residuals [3], thereby being highly abundant in dietary fibers (DFs) [4]. However, DF in WB is dominated by insoluble dietary fiber (IDF), while there is less soluble dietary fiber (SDF), causing deterioration of the texture and other quality properties of food, which hinders its application for the food industry and leads most WB to remain as livestock feed [3,5]. Therefore, techniques to improve the content of SDF in WB are of critical importance to promote value-added applications.

Arabinoxylan (AX) is the main DF in WB, accounting for over 50% of total dietary fiber (TDF) [6]. However, most AXs are embedded in the cell wall matrix and intertwined with other macromolecules, which makes them difficult to extract with water [7]. Water extractable arabinoxylan (WEAX) and water unextractable arabinoxylan (WUAX) differ in both processing and physiological functions [8]. For example, due to its high water holding capacity (WHC), WEAX could improve the viscosity of the dough for bread baking [9]. By reason of the high WHC of WEAX, bran has been reported to have a so-called “bulking” effect to promote gut transit and avoid any constipation [5]. WEAX has also been reported to be more readily fermented by gut microbiota in the large intestine than WUAX, with a well-balanced gut microbiota composition and metabolism [10,11]. In addition, SDF has been associated with lowering cholesterol, glucose, and insulin levels owing to its capacity to bind oil [12]. Based on the above advantages, it is essential to improve WEAX in WB.

Extrusion-cooking is one of the most effective approaches to converting IDF to SDF through a quick thermal procedure [13,14]. During this process, WB undergoes high temperatures, high pressure, and strong shear forces, after which the structure gets loosened and degraded, releasing more nutrient molecules [14]. Prior studies demonstrated that extrusion could improve SDF content, accelerate protein denaturation, starch gelatinization, and degradation, thus modifying the functionalities of flours [15,16]. Meanwhile, during extrusion processing, ester bonds between phenolic compounds (PCs) and other cellular constituents, i.e., DF, partially get hydrolyzed to release more PCs, indicating higher anti-oxidant potential [17]. Furthermore, enzymatic treatment is known as a mild modification method with high hydrolysis specificity and a fast reaction speed [14]. Enzymes such as xylanase can break down β-(1–4)-linkages among xylopyranoside residues, causing WEAX content to increase in WB [7]. More than destroying DF structures, enzyme treatment could also improve the release of WB flavor substances [18].

Until now, although many technologies have been developed to improve DF quality in WB (Table 1), the properties of WEAX obtained are dependent on raw materials and processing conditions [17,19]. Black wheat, in particular, is a distinctive wheat variety compared to normal wheat because of its higher levels of proteins, DFs, PCs, and minerals [20,21,22]. Black wheat also contains a higher content of antioxidants, which can actively remove radicals, reduce oxidative stress-induced cellular damage, and exert anti-aging and anti-inflammatory effects [23]. In addition, compared to WB, black wheat bran (BWB) is more abundant in DFs and PCs [22], yet there is little information available on WEAX and other nutritional properties of BWB during modification [24]. For this purpose, we first optimized conditions for extrusion and enzymatic co-modification to achieve the highest WEAX in BWB. Other nutritional components and physicochemical properties were determined under the selected co-modification. In addition, the in vitro prebiotic potential of the co-modified BWB was also evaluated.

## 2. Materials and Methods

### 2.1. Materials

The black wheat seed was provided by the Cotton Research Institute of the Shanxi Academy of Agricultural Sciences (Taiyuan, China). The xylanase (food grade 50,000 U/g), cellulase (food grade 50,000 U/g), high-temperature α-amylase (food grade 40,000 U/g), and acid protease (food grade 700,000 U/g) were obtained from Solarbio (Beijing Solarbio Technology Co., Ltd., Beijing, China). The α-amylase (15 U/mg) and pepsin (474 U/mg) were purchased from Novozymes (Bagsvaerd, Denmark).

### 2.2. Preparation and Pretreatment of Black Wheat Bran (BWB)

The outer skin of the black wheat seed was first removed by gently rubbing the kernels to avoid lipid rancidity and microbial spoilage. In preparation of BWB, the water content of the kernels was adjusted to 24%, and a Buhler mill (Bühler Group, Uzwil, Switzerland) was introduced to grind the kernels into BWB. The superheated steam drying technique at 280 °C for 20 s was then applied to improve its nutritional integrity, and BWB was passed through a 0.18 mm mesh before use.

### 2.3. Co-Modification of BWB with Extrusion and Different Enzyme Formulas

As a preliminary study, the extrusion conditions were set as follows: moisture content of the bran at 22%; temperature of screw zone I at 55 °C, screw zone II at 100 °C, and screw zone III at 110 °C; rotating speed of 15 Hz; and feeding speed of 13 Hz. A series of enzyme formulas were tested with extrusion, as listed in Table 2.

### 2.4. Single-Factor Experiment Design

To improve the concentration of water extractable arabinoxylan (WEAX) in BWB, single-factor experiments were designed for optimizing factors, including the temperature of screw zone III, moisture content, cellulase concentration, xylanase concentration, and screw speed. The temperature of screw zone I was fixed at 55 °C, screw zone II at 100 °C, feeding speed at 13 Hz, high-temperature α-amylase at 0.4%, and acid protease at 0.4%.

#### 2.4.1. Factor 1: Xylanase Concentration

The concentration of xylanase was set at 0.3%, 0.6%, 0.9%, 1.2%, and 1.4%, respectively. The temperature of screw zone III was fixed at 150 °C, the screw speed at 15 Hz, the water content at 26%, and the cellulase concentration at 0.4%.

#### 2.4.2. Factor 2: Cellulase Concentration

The concentration of cellulase was set at 0.2%, 0.4%, 0.6%, 0.8%, and 1%, respectively. The temperature of screw zone III was fixed at 150 °C, the screw speed at 15 Hz, the moisture content at 26%, and the xylanase concentration at 0.9%.

#### 2.4.3. Factor 3: Temperature of Screw Zone III

The temperature of screw zone III was set at 110 °C, 120 °C, 130 °C, 140 °C, and 150 °C, respectively. The screw speed was fixed at 15 Hz, the moisture content at 26%, the cellulase concentration at 0.6%, and the xylanase concentration at 0.9%.

#### 2.4.4. Factor 4: Screw Speed

The screw speed was set at 13 Hz, 14 Hz, 15 Hz, 16 Hz, and 17 Hz, respectively. The temperature of screw zone III was fixed at 150 °C, the moisture content at 26%, the cellulase concentration at 0.4%, and the xylanase concentration at 0.9%.

#### 2.4.5. Factor 5: Moisture Content of BWB

The moisture content of BWB was set at 18%, 20%, 22%, 24%, and 26%, respectively. The temperature of screw zone III was fixed at 150 °C, the screw speed at 15 Hz, the cellulase concentration at 0.6%, and the xylanase concentration at 0.9%.

### 2.5. Orthogonal Experiment Design

According to the results of the single-factor experiment, an orthogonal experiment including five factors at three levels was designed to determine the optimized co-modification conditions, as shown in Table 3 [L_18_(3^5^)].

### 2.6. Preparation of the Co-Modified BWB

The co-modified BWB was prepared based on the method of Zhang et al., with slight modifications [14]. Following superheated steam drying, the BWB was modified with CXAP using a twin screw extruder (FUMACH MCGS, Hunan Fumaco Food Engineering Technology Co., Ltd., Changsha, China). CXAP was incubated with water at 37 °C for 30 min, and the water volume was calculated to achieve the moisture content for modification, i.e., 22%. The concentrations of xylanase, cellulase, high-temperature α-amylase, and acid protease were 0.9%, 0.6%, 0.4%, and 0.4%, respectively. The parameters of the extruder were set as follows: the temperature of screw zone I was 55 °C, screw zone II was 100 °C, screw zone III was 140 °C, and the feeding speed was 13 Hz.

### 2.7. Determination of Nutritional Components and Physicochemical Properties of BWB

#### 2.7.1. WEAX Content and Other Nutrients

The content of WEAX in BWB was determined by following the orcinol-hydrochloric acid-ferric acid method, as described previously [30]. Soluble dietary fiber (SDF) was determined by using an enzymatic–gravimetric method with the fiber assay kit method [31]. A Megazyme kit for total starch assay (Megazyme International Ireland, Ltd., Wicklow, Ireland) was used for the determination of starch content [30]. Moisture content was determined by drying fresh samples to a constant weight at 105 °C in a hot air oven [32]. Soxhlet extraction techniques were used to determine the crude fat content of BWB [33]. Total phenolic content was determined by the Folin–Ciocalteau method [34].

#### 2.7.2. Water Holding Capacity (WHC)

The WHC was determined based on Jureviciute et al. [31]. A dried sample (3 g) was mixed with overloaded deionized water and allowed to hydrate for 2 h. The excess water was then removed by allowing the wet sample to drain on a fine-meshed wire screen. A portion of the wet sample on the screen was carefully removed, weighed, and dried to a constant weight of ± 0.05 mg in a forced-air oven (110 °C). The *WHC* was calculated as follows:

$$WHC\;=\;\frac{wet\;weight-dry\;weight}{dry\;weight}$$

#### 2.7.3. Oil Holding Capacity (OHC)

The OHC measurement was based on Oh’s method with a slight modification [35]. A dried sample (5 g) was mixed with soybean oil in a centrifugal tube and left for 1 h at room temperature (25 °C). The mixture was then centrifuged at 1500× *g* for 10 min. The supernatant was discarded, and the pellet was recovered by filtration through a nylon mesh. The *OHC* was expressed as follows:

$$OHC\;=\;\frac{pellet\;weight-dry\;weight}{dry\;weight}$$

#### 2.7.4. Cholesterol Adsorption Capacity (CAC)

CAC was determined according to the method described by Liao et al. [36]. The yolk of fresh eggs was separated, and 9 times the weight of distilled water was added to mix it into an emulsion. Twenty grams of dried BWB were mixed with 50 g of yolk emulsion in a triangular flask of 200 mL. The pHs of the system were adjusted to pH 2.0 and pH 7.0, respectively. Afterwards, the system was oscillated for 2 h at 37 °C and centrifuged at 4000 r/min for 10 min (precipitation of BWB). The supernatant was diluted 5 times with 90% acetic acid. The cholesterol content was determined by colorimetry at 550 nm using phthalaldehyde as a chromogenic reagent.

$$A\;=\;\frac{m_{3}-m_{2}}{m_{3}}$$

*A*: cholesterol adsorption capacity;

*m*_1_: weight of wheat bran after drying;

*m*_2_: cholesterol content in the supernatant after adsorption;

*m*_3_: cholesterol content in the supernatant before adsorption.

#### 2.7.5. Microscopic Analysis

The microscopic images of native and co-modified BWB samples were visualized using an FEI/Quanta 450 FEG scanning electron microscope (SEM, Thermo Fisher Scientific, Waltham, MA, USA) based on the method described by Kasipandi and Dang [37]. The samples were placed on a specimen holder with double-sided tape, fixed in studs, and coated with gold (60 s, 5 mA) before observation. The images of bran samples were digitally analyzed using NIS-Elements Software Version 4.20 (Nikon Instruments Inc., Melville, NY, USA).

### 2.8. In Vitro Digestion

In vitro digestion was conducted by following the method of Han et al. [38]. A crushed sample of 12.00 g was dissolved in 200 mL of deionized water. Afterwards, α-amylase was prepared by adding 10.00 mg of α-amylase to 1.25 mL of CaCl_2_ (1 mmol/L, pH 7.0) and incubating it in a shaking water bath at 37 °C for 30 min. The pH of α-amylase solution was adjusted to pH 2.0 by 6 mol/L HCl. Pepsins were prepared by dissolving them in 0.1 mol/L HCl and shaking them in a water bath for 2 h. The pH of the pepsin solution was adjusted to 6.80 with 6 mol/L of NaOH. Trypsin (0.22 g) and bile (0.70 g) were dissolved in Na_2_CO_3_ (0.5 mol/L, 10 mL) and oscillated in a water bath for 4 h. The solution mixture was transferred to a 1 kDa dialysis bag for overnight storage. The dialysis bag was replaced, and dialysis continued for another 2 h. The solution was freeze-dried and preserved to simulate intestinal fermentation.

### 2.9. In Vitro Fermentation and Analysis

#### 2.9.1. Fermentation Medium

The fermentation medium was prepared according to the method of Yu et al. with a slight modification [39]. The medium contained: peptone 3 g/L, yeast 4.5 g/L, tryptone 3 g/L, NaCl 4.5 g/L, KCl 2.5 g/L, K_2_HPO_4_ 0.04 g/L, KH_2_PO_4_ 0.04 g/L, MgSO_4_ 0.01 g/L, CaCl_2_ 0.01 g/L, NaHCO_3_ 2 g/L, L-cysteine 0.5 g/L, bile salt 0.5 g/L, tween 80 2 mL/L, and heme chloride 0.05 g/L.

#### 2.9.2. Fecal Inoculum

Fresh feces were collected from two healthy male and one healthy female volunteers (23–26 years old). The candidates had not received any antibiotic treatment within the last three months or any probiotics before sampling, and no history of intestinal disease was reported. Fecal inoculum was prepared by diluting the feces with pre-reduced PBS (1 mmol/L, pH 7.0) at a ratio of 20%. All materials used in the collection process were sterile.

#### 2.9.3. Anaerobic Fermentation

The method of simulated intestinal fermentation refers to Olano-Martin and is slightly modified [40]. The 72 mL primary culture medium was added to the aseptic fermentation flask, and the pH value was adjusted to 7.0. The 8 mL fecal slurry was added into the fermentation flask containing culture medium, and 29 freeze-dried samples digested in vitro were added according to a 2% ratio. After mixing, the fermentation was carried out in an anaerobic environment at 37 °C. The same volume of fecal slurry was added to the new group. The intestinal flora and short-chain fatty acid content were measured after 24 h of fermentation. The fecal samples were collected and added within 30 min. Each sample was repeated twice.

#### 2.9.4. Determination of Bifidobacterium and Lactobacillus

DNA extraction of fermentation microbiota was carried out with a bacterial genomic DNA rapid extraction kit [41]. Real-time qPCR was used to detect the content of *Bifidobacterium* and *Lactobacillus* in the fermentation broth. PCR primers were designed based on the 16S rDNA gene sequences of *Bifidobacterium* and *Lactobacillus* [42]. The primer sequences of *Bifidobacterium* are 5′-TCGCGTCCGGTGTGAAAG-3′ and 5′-CCACATCCAGCATCCAC-3′. The primer sequences of *Lactobacillus* are 5′-AGCAGTAGGGAATCTTCCA-3′ and 5′-CACCGCTACACATGGAG-3′. The reaction procedure was: 50 °C enzyme activation for 2 min, 95 °C pre-denaturation for 10 min, and 40 cycles (95 °C deformation for 15 s, 60 °C for 1 min) [43]. The number of *Bifidobacterium* and *Lactobacillus* in the samples was analyzed according to the Ct values.

#### 2.9.5. Determination of Short-Chain Fatty Acids (SCFAs) and pH Values

Gas chromatography (GC) was used to measure the production of SCFAs during fermentation [44]. The fermentation digest was incubated with a 25% metaphosphate solution for 30 min and then centrifuged at 10,000 rpm for 20 min. The obtained supernatant was collected and filtered by a 0.45 μm aqueous membrane. A 25 μL aliqout was injected into the DB-FFAP (15 mm × 0.32 mm × 0.25 um) chromatographic column. The temperature profile was from 100 °C to 120 °C at 2 °C/min, then maintained at 120 °C for 10 min.

The pH value of the fermentation digest was determined by a pH meter (Dolly Scientific Instrument Co., Ltd., Guangzhou, China).

### 2.10. Statistical Analysis

The results were analyzed using SPSS 21.0 statistical software (SPSS Inc., Chicago, IL, USA). Values are expressed as means ± standard deviation. All treatments were performed in three parallel experiments, and each sample was analyzed at least in triplicate. The data were analyzed using repeated measures analysis of variance with the Duncan test. A significant difference was defined as *p* < 0.05.

## 3. Results and Discussion

### 3.1. Co-Modification by Extrusion and CXAP Increases WEAX Content in BWB

Arabinoxylan (AX) is an important dietary fiber source in BWB, which is dominated by water unextractable arabinoxylan (WUAX) and has less water extractable arabinoxylan (WEAX) [45]. In this study, the content of WEAX in native BWB was only 0.312 ± 0.04 g/100 g, which can be seen from Table 4. With extrusion treatment, WEAX content was slightly increased by 66% (*p* < 0.05). Whereas a much higher increase in WEAX was observed with WB in a recent study conducted by Demuth et al. That is, WEAX was almost triplicated by extrusion [46], while there was only 0.093 ± 0.04 g/100 g of WEAX in native WB. The difference might be explained by different binding tightnesses in the cell wall matrix, also indicating a species difference [17].

Co-modification of BWB by enzymes and extrusion showed a larger impact on the content of WEAX than with only extrusion treatment, but this was constrained by enzyme type and formula [47,48]. Cellulase and xylanase are both cell wall-degrading enzymes [49]. They significantly increased WEAX to a similar level of 0.818 ± 0.04 and 0.912 ± 0.05 g/100 g (*p* < 0.05), respectively. A much higher level of WEAX, 1.183 ± 0.04 g/100 g (*p* < 0.05), was observed when these two enzymes were combined. Interestingly, although the formula of dual-enzyme thermostable α-amylase and acid protease only reached a similar level to that of WEAX in the extrusion group, i.e., 0.592 ± 0.02 g/100 g, the content was still higher than in native BWB. Considering the nutrients released by different types of enzymes [18], the above four enzymes, CXAP, were combined with extrusion, and the highest WEAX content was achieved in a formula of 1.210 ± 0.05 (*p* < 0.05).

### 3.2. Analysis of the Single-Factor Experiment and the Orthogonal Experiment

**Figure 1:**
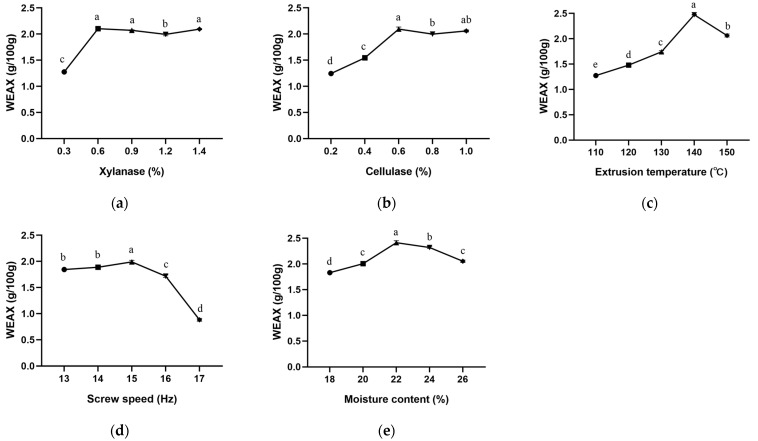
The effects of enzyme concentrations and extrusion parameters on the content of WEAX. (**a**) Xylanase; (**b**) Cellulase; (**c**) Extrusion temperature; (**d**) Screw speed; (**e**) Moisture content. Different letters in the same column indicate that there was a significant difference among different treatments (*p* < 0.05, *n* = 3).

The effects of single enzyme xylanase and cellulase concentrations on WEAX content are shown in Figure 1a,b, respectively. WEAX content kept increasing until the xylanase concentration reached 0.6% of the substrate and remained stable at around 2 g/100 g afterwards. A similar optimum enzyme concentration of 0.6% was also seen for cellulase. This could be explained by the fact that the binding sites for specific enzymes are limited when the amount of substrate is fixed [50]. A previous study using cellulase and hemicellulases also released more SDF from IDF than the native group of millet [10].

There was a significant effect of extrusion temperature on WEAX content, as shown in Figure 1c. The initial value of WEAX was only 1.27 g/100 g at 110 °C, and it increased to a peak level of 2.47 g/100 g when the temperature came to 140 °C, followed by a decline to 2.06 g/100 g until the highest temperature of 150 °C was reached. A similar result was obtained by Andersson et al., who found that the AX extractability of WB was significantly increased from 90 °C to 130 °C [3]. Higher temperatures may assist in breaking the glycosidic bonds between IDF substances [14]. Here we demonstrate that an appropriate temperature may be necessary for thermal modification.

Different from other parameters, with the increase in screw speed from 13 Hz to 15 Hz (Figure 1d), the content of WEAX increased slightly from 1.84 g/100 g to 1.99 g/100 g (*p* < 0.05), while there was a decrease to 0.88 g/100 g when screw speed increased to 17 Hz. This is different from the result of Fadel et al., who reported a tendency for WEAX content in defatted rice bran to increase with extrusion screw speed from 80 rpm to 160 rpm [6]. A higher extrusion speed will exert high shear force to liberate ferulic acid side chains esterified to AX residues, which might induce higher exposure of AX to water, thus increasing WEAX content [51]. However, the effect of extrusion speed might influence extrusion force and reaction time with the substrate. The extrusion force will increase as the extrusion screw speeds up, while the reaction time will decrease, which may result in a decrease in WEAX [6], indicating an optimum speed requirement. In this study, we demonstrate a screw speed of 13 Hz for BWB WEAX extraction.

Moisture content showed a relatively less pronounced effect on WEAX (Figure 1e) compared to the above-mentioned conditions. It was found that as the moisture content of BWB increased, the WEAX content in BWB increased slightly from 1.83 g/100 g to 2.41 g/100 g (*p* < 0.05). Nevertheless, when the moisture content was greater than 22%, the WEAX content decreased slightly to 2.05 g/100 g (*p* < 0.05). Our results demonstrate a suitable moisture content for BWB co-modification [52]. Under the high temperature and shear force during extrusion, the moisture contained in BWB will be evaporated and squeezed out, leaving the original locations empty to achieve the puffing effect [53]. However, when the moisture content is low, the extruded BWB may be relatively dense, which may lower the physicochemical properties, such as water and oil holding capacity [16]. If the moisture content is above the suitable level, the extrusion temperature may be lowered, which will weaken the thermal modification ability of extrusion.

According to WEAX contents from the single-factor experiments, we designed the above orthogonal experiment (Table 5). According to the value of range, the importance order of the co-modification conditions for WEAX content was: temperature of screw zone III, xylanase, cellulase, moisture content, and screw speed. Thus, the optimized conditions for co-modification of BWB were: temperature of screw zone III at 140 °C; xylanase at 0.9%; cellulase at 0.6%; moisture content at 22%; and screw speed at 16 Hz. The highest WEAX content was 3.16 ± 0.03 g/100 g. The co-modification conditions of BWB were verified, and a similar WEAX of 3.03 g/100 g was obtained (*p* > 0.05), which was over 9 times higher than the native BWB.

### 3.3. Distinct Alteration of Nutrients and Physicochemical Properties of BWB under Co-Modification

The fat, starch, and protein contents of BWB in native bran were 3.19 g/100 g, 18.47 g/100 g, and 19.32 g/100 g, respectively (Table 6). With co-modification by extrusion and CXAP, the content of fat and starch was significantly lower than the native control, with a decline of 13.17% and 12.07% (*p* < 0.05), respectively. A similar tendency was obtained by Ye et al. [13], who reported that extrusion induced a reduction in fat and starch content by 17% and 10%, respectively. The decrease in fat content might be due to unsaturated fatty acid oxidation caused by extrusion at high temperatures [54]. Additionally, high temperature and pressure during the extrusion process may induce starch gelatinization and degradation, leading to the formation of lipid-starch complexes [55,56]. Meanwhile, high-temperature α-amylase in CXAP may assist in starch degradation. Interestingly, the protein content here remained unchanged, indicating the high quality of BWB through current co-modification methods.

High-phenolic compounds (PCs) are known for their anti-oxidative activity and cholesterol-lowering properties [57]. Co-modification of BWB with extrusion and CXAP significantly increased TPC by 9% (*p* < 0.05), corresponding to the results as shown by Ye et al. in WB [13]. Patil et al. also reported a threefold increase in TPC by extrusion in composite wheat bread substituted with 30% finger millet [15]. There are few reports in support of the effect of the addition of enzymes on releasing TPC [58], so the increasing effect might come from the extrusion treatment. Extrusion could assist in breaking down the ester- or ether-bounds between PC and cell wall polysaccharides to release especially bound phenolics and increase free phenolics [59].

BWB is a high-quality DF source, with 41.97% of the native control. The percentage was significantly elevated by 10% to a level of 46.04% upon co-modification with extrusion and CXAP (*p* < 0.05). The initial content of SDF was only 2.43%, and co-modification almost doubled the SDF content (*p* < 0.05). The result is consistent with the tendency reported by Zhang et al. through co-modification by semi-solid enzymatic hydrolysis and extrusion, but improved to a much higher extent [14]. The increase in TDF might be attributed to the creation of resistant starch during extrusion [60]. Extrusion here may loosen the spatial structure of DF, thus providing more binding sites for CXAP hydrolysis and allowing more conversion of IDF to SDF [61,62].

Through modification of DF and an increase in SDF yield, the physicochemical properties and functional activity of BWB, such as water holding capacity (WHC), oil holding capacity (OHC), and cholesterol adsorption capacity (CAC), may be improved [63]. As shown in Table 5, the WHC and OHC of BWB were significantly increased by 100% and 71%, respectively, with co-modification (*p* < 0.05). Similar findings were obtained by Li et al. and Ma et al. [63,64]. WHC and OHC stand for the amount of water and oil that can be retained by DF, which is influenced by surface area and particle sizes [65]. Co-modification treatments destroy glycosidic bonds in DF, resulting in the exposure of more hydrophilic or lipophilic groups and a more porous structure, which finally enhances water or oil retention capacity [66]. With higher oil retention, the CAC of BWB was improved, as expected. Although co-modification significantly increased the CAC of BWB under both neutral and acidic conditions, the CAC under pH 7.0 was much higher than that under pH 2.0, implying a stronger cholesterol-lowering effect in the small intestine than in the stomach [67]. The increase in TPC above may also be responsible for an elevated CAC [57].

### 3.4. Physicochemical Structure

**Figure 2:**
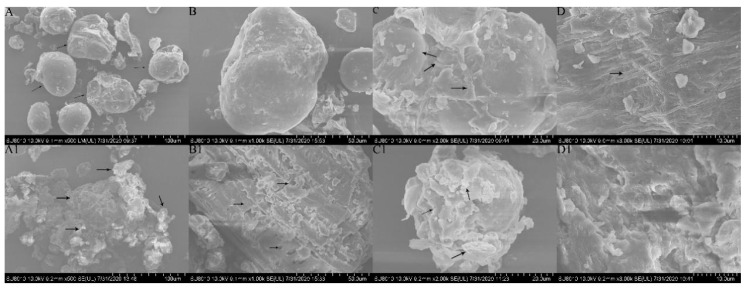
SEM of BWB before (**A**–**D**) and after (**A1**–**D1**) co-modification. A (**A1**), B (**B1**), C (**C1**), and D (**D1**) were observed under a scanning electron microscope at 500, 1 K, 2 K, and 3 K times, respectively.

To better understand the mechanism of enhanced WHC, OHC, and CAC of BWB after co-modification, the physicochemical structure of the bran was observed using scanning electron microscopy (SEM). SEM micrographs of BWB were magnified 500 (A), 1000 (B), 2000 (C), and 3000 (D) times, as shown in Figure 2. The microstructure of the native BWB granules was round, smooth, and compact, while co-modified BWB (A1, B1, C1, D1) showed breakage and dissociation and became looser and more porous, with a rough surface. Similar results were observed using B. natto-fermented millet bran [65]. As mentioned above, conversion of IDF to SDF and breakage of glycosidic bonds by extrusion and enzymatic degradation might be the main reasons for the rough surface structure [50,66]. Thus, WHC, OHC, and CAC were enhanced with the improved structure.

### 3.5. Inulin, BWB, and Co-Modified BWB Fermentation Increase the Relative Abundance of Bifidobacterium and Lactobacillus

Next, we investigated the prebiotic potential of co-modified BWB by evaluating the abundance of *Bifidobacterium* and *Lactobacillus* using simulated intestinal fermentation. The group without any carbohydrates served as the negative control. Fermentation with widely applied prebiotic inulin was taken as the positive control.

**Figure 3:**
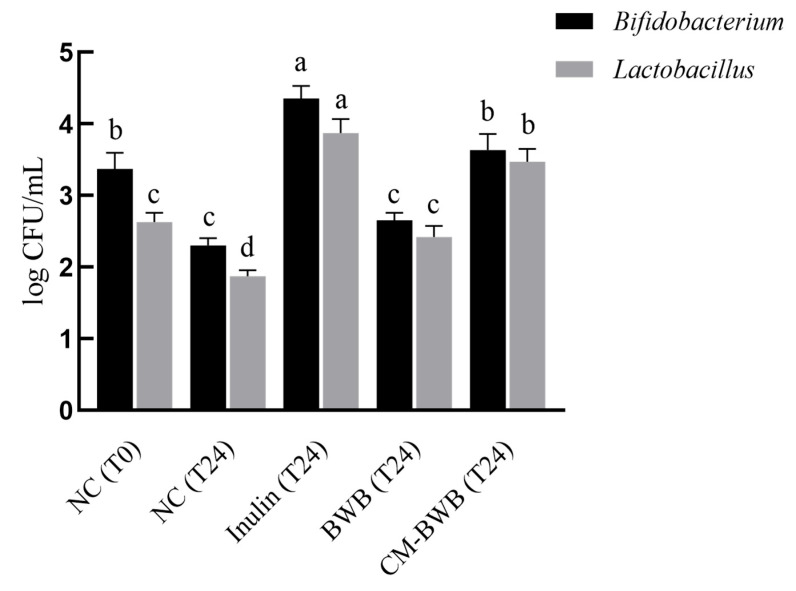
Changes in *Bifidobacterium* and *Lactobacillus* abundance in the fermentation broth. Note: Different letters indicated that there was a significant difference among different treatments (*p* < 0.05, *n* = 3).

In the negative control (NC), the abundances of *Bifidobacterium* and *Lactobacillus* were significantly decreased by 32% and 29%, respectively (*p* < 0.05) from T0 to T24 (Figure 3), indicating the necessity of carbon sources for the growth of beneficial bacteria [68]. Fermentation with inulin at T24 almost doubled the abundance of *Bifidobacterium* and *Lactobacillus* compared to the NC, corresponding to the observations of Feng et al. and Wang et al. [67,69]. BWB significantly increased the abundance of *Lactobacillus*, but to a lesser extent, with only 29% higher than the NC at T24 (*p* < 0.05). Compared to native BWB, co-modified BWB resulted in higher abundances of *Bifidobacterium* and *Lactobacillus* as compared to inulin at T24, suggesting the potential for exploitation as prebiotics. Stimulation of beneficial bacteria growth here might be explained by the higher SDF content and, more specifically, the higher content of WEAX in co-modified BWB. Correlated to the SEM micrographs in Figure 2, the loose, porous, and rough surface may provide a higher chance of being utilized by the bacteria [65]. Evidence can be found in the results reported by Demutu et al. that extruded and milled WBAX stimulated faster *Bifidobacteria* growth than the native control [70].

### 3.6. Changes of SCFA Content and pH in Fermentation Broth In Vitro

Along with the improvement of colonic beneficial bacteria, co-modification of BWB could also regulate gut microbial metabolism products, which will reduce colonic pH values and further improve beneficial bacteria [70]. Here, the content of short-chain fatty acids (SCFAs: acetic acid, propionic acid, and butyric acid) and branched SCFAs (isobutyric acid and isovaleric acid) at T0 in the NC was only 1.21, 0.62, 0.15, 0.031, and 0.025 mmol/L, respectively (Table 7). After 24 h of fermentation, we observed a significant increase in undesired fermentation products, isobutyric acid and isovaleric acid, to 0.26 and 0.49 mmol/L, corresponding to the reduced *Bifidobacterium* and *Lactobacillus*, respectively. When carbon sources, especially DF, are lacking, microorganisms will initiate amino acid fermentation, resulting in higher production of undesired isobutyric acid and isovaleric acid [71].

Inulin was more readily fermented than BWB and induced a much higher production of acetic acid, with a 65% increase, while producing less propionic acid and butyric acid. Acetic acid is the most abundant SCFA in gut fermentation, serving as the intermediate molecule for the synthesis of butyric acid [72], which may imply the already existing conversion of acetic acid to butyric acid in BWB. However, isobutyric acid and isovaleric acid production were 82% and 2 times higher in BWB fermentation than inulin, respectively, indicating possible amino acid fermentation [71]. In addition, inulin fermentation obtained higher total SCFAs than the NC and BWB, consistent with the pH value tendency.

Interestingly, fermentation of co-modified BWB dramatically increased butyric acid content compared to the NC, which was 7 times and 78% higher than inulin and BWB fermentation, respectively. Butyric acid is an important energy source for intestine epithelial cells to improve the stability of intestinal barrier function, indicating the potential of co-modified BWB to strengthen gut barriers and lower the occurrence of colorectal carcinoma [73]. Although the content of acetic acid in co-modified BWB was slightly lower than in inulin fermentation, it was 42% higher than BWB fermentation. The branched SCFA isobutyric acid was significantly reduced with co-modification of BWB, and a much higher total SCFA was obtained with co-modification, corresponding to a higher abundance of *Bifidobacterium* and *Lactobacillus* and a lower pH value. All these results observed may be attributed to the higher WEAX in BWB with co-modification, which made it more readily accessible by gut microbiota to produce more SCFAs and a lower pH, in turn feeding beneficial bacteria and improving gut health [74].

## 4. Conclusions

In summary, we show that co-modification of extrusion and enzymes could dramatically increase WEAX content in BWB. However, the effects were dependent on extrusion conditions, including temperature, moisture content, screw speed, and enzyme formulas. Other nutrients, especially phenolic compounds and soluble dietary fiber were more readily released under extrusion and enzymatic hydrolysis. Meanwhile, co-modified BWB displayed better physicochemical properties, as evidenced by higher water holding capacity, oil holding capacity, cholesterol adsorption capacity, and a more porous microstructure under a scanning electron microscope. Notably, fermentation of co-modified BWB achieved a similar abundance of beneficial bacteria as compared to the widely applied inulin, with a much higher level of butyric acid production. This knowledge may contribute to improving technologies to modify BWB and extending co-modified BWB’s application as a high-SDF cereal source.

## Figures and Tables

**Table 1:** Comparison of different modification methods of wheat bran and their functional properties.

Raw Material	Modification Method	Modification Conditions	Physicochemical Functions	Biological Functions	References
Wheat bran	Ultra-sonication	Time: 0.5 h, 1.0 h, 2.0 h; Power: 200 w, 400 w, 600 w	Molecular weight and viscosity of AX↓;	None	[25]
	Enzymatic (xylanase)	Time: 5 min, 10 min, 15 min; Xylanase content: 1 mg/L, 5 mg/L, 10 mg/L	Solubility and degree of branching↑	None	
	Trifluoroacetic acid (TFA)	Time: 0.5 h, 1.0 h, 2.0 h; TFA content: 0.025 M, 0.05 M, 0.075 M	Solubility and degree of branching↑	None	
Crude wheat bran	Microwaving	Time: 1 min; Power: 1680 W	Folic acid↑, Vitamin B2↓, WEAX↑, TPC↑, Ferulic acid↑,	ABTS radical scavenging capacity↑	[13]
	Steam	Time: 10 min; Temperature: 100 °C	Folic acid↑, Vitamin B2↓, TPC↑, Ferulic acid↑	None	
	Extrusion	Temperature: 60 °C, 90 °C, 120 °C, 150 °C Moisture content: 17 g/100 g; Screw speed: 275 rpm	Folic acid↑, Vitamin B2↓, WEAX↑, Swelling force↑, TPC↑, Ferulic acid↑,	Oxygen radical absorbance capacity↑	
	Superheated steam	Temperature: 220 °C; Time: 90 s	Folic acid↑, Vitamin B2↓, TPC↑, Ferulic acid↑	None	
	Fermentation (yeast)	Temperature: 37 °C; Time: 24 h	SDF↑, WEAX↑, TPC↑, Ferulic acid↑	None	
	Enzymatic treatment (xylanase)	pH: 5.0	Folic acid↑, Vitamin B2↓, TPC↑, Ferulic acid↑,	DPPH radical scavenging capacity↓	
Wheat bran	Fermentation (yeast and lactic acid bacteria)	Temperature: 37 °C; Time: 24 h; Moisture content: 50%	WEAX↑, TDF↑, SDF↑, Phytic acid↓, Protein↓, WHC↑, Water Retention Capacity↑	None	[26]
Coarse wheat bran	Micronization	Screw speed: 960 rpm; Power: 25 Hz, 50 Hz	SDF↑, Ferulic acid↑, TPC↑, ABTS and DPPH radical scavenging capacity↑, Water Retention Capacity↓, IDF↓	None	[27]
Wheat bran	Extrusion	Moisture content: 24%; Screw speed: 400 rpm; Temperature: 130 °C	AX extractability↑, TDF-, Total AX-, β-glucan extractability↑	None	[3]
Coarse wheat bran	Extrusion and enzymatic hydrolysis	Screw speed: 100 rpm, 200 rpm; Moisture content: 12%, 14%, 16%; Temperature: 105 °C, 120 °C, 135 °C Enzymatic hydrolysis time: 0, 30, 60, 120 min; Temperature: 45 °C	Solubility of fiber↑, Water Binding Capacity↓, Microstructure disruption	None	[28]
Wheat bran	Steam explosion	Pressure: 0.3 MPa, 0.5 MPa, 0.8 MPa; Time: 5 min	Total flavonoids↑, TPC↑, SDF↑,Fatty acid value↓, Peroxide value↓, IDF↓, Rancidity↓	DPPH free radical scavenging activity↑	[29]

↑: Increasing, ↓: Decreasing, -: Stable.

**Table 2:** Overview of the enzyme formulas tested with extrusion.

Enzyme Formula	Concentration (%)
Enzyme-free	0
Cellulase	0.4
Xylanase	0.9
Cellulase, Xylanase	0.4, 0.9
High-temperature α-amylase, Acid protease	0.4, 0.4
CXAP ^1^	0.6, 0.9, 0.4, 0.4

^1^ CXAP: cellulase, xylanase, high-temperature α-amylase, and acid protease mixture.

**Table 3:** Orthogonal experiment design for co-modification of BWB.

Level	Factor				
	Xylanase (%)	Cellulase (%)	Temperature of Screw Zone III (°C)	Screw Speed (Hz)	Moisture Content (%)
1	0.3	0.4	130	14	20
2	0.6	0.6	140	15	22
3	0.9	0.8	150	16	24

**Table 4:** Effects of different co-modifications on the WEAX content of BWB.

Method	Enzyme	WEAX (g/100 g)
Control	None (Native)	0.312 ± 0.04 ^d^
	None (Extrusion)	0.519 ± 0.05 ^c^
Co-modification by extrusion with a single enzyme	Cellulase	0.818 ± 0.04 ^b^
	Xylanase	0.912 ± 0.05 ^b^
Co-modification by extrusion with a dual enzyme	Cellulase, Xylanase	1.183 ± 0.04 ^a^
	Thermostable α-amylase, Acid protease	0.592 ± 0.02 ^c^
Co-modification by extrusion with CXAP	Cellulase, Xylanase, Thermostable α-amylase, Acid protease	1.210 ± 0.05 ^a^
Co-modification by extrusion with a single enzyme	Cellulase	0.818 ± 0.04 ^b^

Note: Different letters in the same column indicate that there was a significant difference among different treatments (*p* < 0.05, *n* = 3).

**Table 5:** Orthogonal experiment for co-modification of BWB.

	A	B	C	D	E	WEAX Content (g/100 g)
1	0.3	0.4	130	14	20	1.68 ± 0.07
2	0.6	0.6	130	15	22	1.41 ± 0.05
3	0.9	0.8	130	16	24	1.30 ± 0.04
4	0.6	0.4	140	15	20	2.01 ± 0.06
5	0.9	0.6	140	16	22	3.16 ± 0.03
6	0.3	0.8	140	14	24	1.84 ± 0.05
7	0.3	0.6	150	16	20	2.70 ± 0.06
8	0.6	0.8	150	14	22	2.13 ± 0.02
9	0.9	0.4	150	15	24	2.76 ± 0.07
10	0.9	0.8	130	15	20	1.35 ± 0.03
11	0.3	0.4	130	16	22	1.57 ± 0.05
12	0.6	0.6	130	14	24	1.20 ± 0.04
13	0.9	0.6	140	14	20	1.84 ± 0.04
14	0.3	0.8	140	15	22	2.38 ± 0.02
15	0.6	0.4	140	16	24	2.20 ± 0.06
16	0.6	0.8	150	16	20	1.41 ± 0.03
17	0.9	0.4	150	14	22	2.68 ± 0.07
18	0.3	0.6	150	15	24	2.06 ± 0.03
K1	1.42	1.83	2.15	2.04	1.89	None
K2	2.24	2.22	2.06	1.73	2.00	None
K3	2.29	1.89	1.74	2.18	2.06	None
Range	0.87	0.39	0.41	0.46	0.16	None

A: Xylanase (%), B: Cellulase (%), C: Temperature (°C), D: Screw speed (Hz), E: Moisture content (%).

**Table 6:** Effects of co-modification of BWB on other nutrients and physicochemical properties.

Index	Native	Co-Modification
Fat (g/100 g)	3.19 ± 0.10 ^a^	2.77 ± 0.06 ^b^
Starch (g/100 g)	18.47 ± 0.20 ^a^	16.24 ± 0.33 ^b^
Protein (g/100 g)	19.32 ± 0.51 ^a^	19.37 ± 0.12 ^a^
TP (mg/g)	2.83 ± 0.18 ^b^	3.09 ± 0.20 ^a^
TDF (g/100 g)	41.97 ± 3.25 ^b^	46.04 ± 3.05 ^a^
SDF (g/100 g)	2.43 ± 0.13 ^b^	8.45 ± 0.22 ^a^
WHC (g/g)	3.12 ± 0.13 ^b^	6.25 ± 0.21 ^a^
OHC (g/g)	4.82 ± 0.18 ^b^	8.24 ± 0.26 ^a^
CAC (pH = 2.0, mg/g)	7.31 ± 0.23 ^b^	16.87 ± 0.35 ^a^
CAC (pH = 7.0, mg/g)	23.63 ± 0.84 ^b^	55.16 ± 1.12 ^a^

Note: Different letters in the same column indicate that there was a significant difference among different treatments (*p* < 0.05, *n* = 3).

**Table 7:** Determination of SCFA content in in vitro fermentation broth.

	SCFA (mmol/L)					pH
	Acetic Acid	Propionic Acid	Butyric Acid	Isobutyric Acid	Isovaleric Acid	
C0	1.21 ± 0.05 ^e^	0.62 ± 0.01 ^d^	0.15 ± 0.01 ^d^	0.031 ± 0.002 ^e^	0.025 ± 0.001 ^e^	7.83 ± 0.03 ^a^
C24	11.31 ± 0.13 ^d^	0.73 ± 0.01 ^c^	0.19 ± 0.02 ^d^	0.260 ± 0.02 ^a^	0.49 ± 0.03 ^a^	7.05 ± 0.07 ^b^
Inulin	47.43 ± 0.76 ^a^	2.67 ± 0.05 ^b^	0.29 ± 0.01 ^c^	0.057 ± 0.001 ^d^	0.037 ± 0.003 ^d^	4.76 ± 0.02 ^e^
BWB	28.67 ± 0.85 ^c^	3.09 ± 0.03 ^a^	1.31 ± 0.01 ^b^	0.104 ± 0.002 ^b^	0.109 ± 0.002 ^b^	6.25 ± 0.02 ^c^
CM-BWB	40.93 ± 0.49 ^b^	2.62 ± 0.03 ^b^	2.33 ± 0.01 ^a^	0.072 ± 0.001 ^c^	0.097 ± 0.001 ^bc^	5.11 ± 0.06 ^d^

C0: Control 0 h; C24: Control 24 h; In: Inulin; CM: Co-modified. Note: Different letters in the same column indicated that there was a significant difference among different treatments (*p* < 0.05, *n* = 3).

## Data Availability

Data is contained within the article.
